# Repeated Inhalation of Peppermint Essential Oil Improves Exercise Performance in Endurance-Trained Rats

**DOI:** 10.3390/nu15112480

**Published:** 2023-05-26

**Authors:** Wei Zhang, Rongpei Shi, Tian Gao, Yang Hu, Jiaheng Zhou, Chenhan Li, Panpan Wang, Hongyan Yang, Wenjuan Xing, Ling Dong, Feng Gao

**Affiliations:** 1 Key Laboratory of Aerospace Medicine, Ministry of Education, School of Aerospace Medicine, Fourth Military Medical University, Xi’an 710032, China; zw1475727965@163.com (W.Z.); rpshi@fmmu.edu.cn (R.S.); yanghu97@fmmu.edu.cn (Y.H.); jiaheng@fmmu.edu.cn (J.Z.); yhy2013@fmmu.edu.cn (H.Y.); wjxing@fmmu.edu.cn (W.X.); fgao@fmmu.edu.cn (F.G.); 2 School of Life Science, Northwest University, Xi’an 710068, China; 3 Division of Health Management, Tangdu Hospital, Fourth Military Medical University, Xi’an 710038, China; tgao@fmmu.edu.cn

**Keywords:** peppermint essential oil, exercise-induced fatigue, redox biomarkers, exercise performance

## Abstract

Peppermint essential oil, being natural and safe, with antioxidant and anti-inflammatory properties, has long been a research interest in relieving fatigue and improving exercise performance. However, the related studies report controversial results, and the mechanisms remain unclear. Here we found that inhalation of peppermint essential oil significantly extended the exhaustion time in rats subjected to 2-week weight-bearing swimming training. Sprague-Dawley rats were subjected to a 2-week weight-loaded forced swimming regimen. Prior to each swimming session, the rats were administered peppermint essential oil via inhalation. An exhaustive swimming test was performed at the end of the protocol. Rats treated with essential oil had significantly extended time to exhaustion compared with exercised rats without essential oil treatment. In addition, treated rats also showed reduced oxidative damage induced by endurance exercise. Notably, the rats receiving two-week essential oil inhalation while not subjected to swimming training did not show improved exercise performance. The findings demonstrate that repeated inhalation of peppermint essential oil enhances the effects of endurance training and improves exercise performance partially by preventing oxidative damage.

## 1. Introduction

Regular physical exercise, including strength and endurance exercise, is believed to improve health and physical fitness. Vigorous or excessive physical activity may induce fatigue, which, if not relieved timely, may have adverse physiological and psychological effects, compromising overall health and decreasing the motivation to exercise. The underlying mechanisms of physical exercise-induced fatigue have drawn the attention of researchers with the aim of searching for effective solutions to alleviate fatigue and enhance exercise performance. It is a general belief that physical exercise challenges the body’s homeostasis and elicits a measurable physiological response. Notably, there is a substantial increase in muscle metabolism, which triggers an increase in the blood circulatory system and gas exchange. Prolonged exercise may result in the excessive consumption of energy, production and accumulation of metabolites, and oxidative damage. Although the underlying mechanisms of chronic fatigue remain unclear, oxidative stress, as well as inflammatory injury, are reported to play important roles in muscle fatigue or peripheral fatigue [1,2]. Proinflammatory cytokines released into the blood circulation by contracting muscles, along with metabolites, especially during endurance exercise, subsequently have numerous effects on other organs, including the brain, causing increased sensations of fatigue, known as central fatigue [3,4].

There is a growing interest in identifying safe and effective bioactive natural products to alleviate exercise-induced fatigue and improve exercise performance. A number of natural bioactive flavonoids found in food-based plants have been identified, which possess the ability to alleviate exercise-induced fatigue via multiple mechanisms, including enhancing muscle strength, regulating energy metabolism, inhibiting inflammatory responses, reducing reactive oxygen species, increasing antioxidant capacity, and regulating neurotransmitter levels [5,6,7]. Additionally, aromatic essential oils have been reported as having physiological effects, such as promoting blood circulation and eliminating muscle pain, as well as psychological effects, including reducing anxiety and relieving stress, thereby eliminating physical and mental fatigue. In particular, peppermint essential oil has garnered significant interest due to its versatile properties. Its major components include menthol, menthone, and 1,8-cineole. Menthol has been shown to have antioxidant, radical-scavenging, anti-inflammatory and pain-relieving properties [8,9]. In addition, menthol and menthone are neuroactive and mind-refreshing, which can facilitate the elimination of mental fatigue [10]. 1,8-cineol has been reported to have anti-fatigue effects related to lactic acid clearance and reduced lactate dehydrogenase and creatine kinase concentrations [11]. Those active ingredients of peppermint essential oil also work synergistically to produce an apparent ambulation-promoting effect to prevent mental fatigue [12]. Peppermint essential oil, with its active ingredients, is believed to reduce oxidative stress and inflammation induced by exercise and thus relieve exercise-induced fatigue and improve exercise performance [10,13]. However, the evidence regarding the anti-fatigue effects of peppermint essential oil remains limited and controversial, with most studies focusing on the immediate effects on exercise performance and its antioxidant and anti-inflammatory activity in reducing exercise-induced oxidative stress.

In this study, we investigated the chronic effects of repeated peppermint essential oil inhalation on endurance exercise performance in rats subjected to 2-week weight-loaded swimming. We found that the rats treated with repeated peppermint essential oil showed significantly extended time to exhaustion in exhaustive swimming tests compared with untreated exercised rats.

## 2. Materials and Methods

### 2.1. Animals

**Figure 1:**
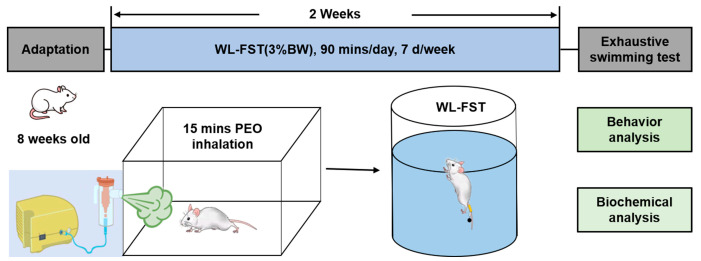
The experimental protocol to investigate the effects of peppermint essential oil inhalation on exercise performance. PEO: peppermint essential oil; WL-FST: weight-loaded forced swim training.

Eight-week-old Sprague-Dawley (SD) male rats weighing 230–280 g were provided by the Experimental Animal Center of Fourth Military Medical University. The rats were placed in an independently ventilated animal cage (BCR-RI01-PEI, Shandong Xinhua Medical Instrument Co., Ltd., Zibo, China) in an environment with a temperature of 22 ± 2 °C and humidity of 50–60% and fed an ad libitum standard diet. The health and behavior of rats were kept careful monitoring on a daily basis to ensure the animals were in a state of normal health and able to freely engage in activities, such as eating, drinking, and moving. After 3-day adaptive feeding and environmental acclimatization, the rats were randomly divided into a control group (saline vehicle, control), peppermint essential oil inhalation for 2 weeks (PEO), chronic fatigue induced by 2-week weight-loaded forced swimming training (WL-FST), and chronic fatigue with peppermint essential oil treatment (WL-FST+PEO), using completely randomized design (n = 8–12/group). Rats in PEO and WL-FST+PEO groups received inhalation of peppermint essential oil (mixed with saline) using a compressed air atomizer (403 M, Jiangsu Yuyue Medical Equipment Co., Ltd., Yancheng, China) for 15 min daily before exercise training, while rats in the control and WL-FST groups received inhalation of saline. Before the experiment, all rats were subjected to 20 min of swimming per day for 3 days. All the animal care and experimental procedures were approved by the Laboratory Animal Welfare and Ethics Committee of Fourth Military Medical University (Approval No.: IACUC—20201051) in compliance with the Guide for the Care and Use of Laboratory Animals published by the US National Institutes of Health. The experiment protocol is illustrated in Figure 1.

### 2.2. Materials and Chemicals

Peppermint essential oil was purchased from Doterra Trading Co., Ltd., Shanghai, China (Cat # 192398, country of origin: USA).

The test kits for glucose (GLU), blood urea nitrogen (BUN), lactic acid (LA), and lactate dehydrogenase (LDH) in serum; superoxide dismutase (SOD), malondialdehyde (MDA), glutathione (GSH), and glutathione peroxidase (GSH-PX) in skeletal muscle tissue; and MDA, GSH, GSH-PX, xanthine oxidase (XOD), and catalase (CAT) in liver tissue, were purchased from Nanjing Jiancheng Bioengineering Research Institute.

### 2.3. Gas Chromatography-Mass Spectrometry (GC-MS) Analysis

The composition and content of peppermint essential oil were analyzed by the Modern Analysis Center of Nanjing University qualitatively and quantitatively by gas chromatography–mass spectrometry (QP-2020, Shimadzu Company, Kyoto, Japan).

Gas chromatographic conditions: DB-5MS capillary column (30 mm × 0.25 mm × 0.25 μm). The initial column temperature was set to 60 °C, maintained for 1 min, and increased to 280 °C at 6 °C/min. The carrier gas was helium, and the carrier gas flow rate was 1.0 mL/min; the split ratio was 120:1, and the injection volume was 0.2 μL. Mass spectrum conditions are as follows: ionization mode—EI; power energy—70 ev; electron source temperature—220 °C; scanning range—m/z, 20–500 amu. The percentage of each component in peppermint essential oil was calculated by normalization according to the GC peak area. NIST2017 spectral library retrieval, standard comparison, and qualitative analysis combined with retention index were used.

### 2.4. Determination of Peppermint Essential Oil Dosage

We first conducted an acute forced swimming experiment to determine the optimal dose of essential oil. Different doses of peppermint essential oil (4, 40, and 400 μL/h) were administered to examine the acute effects of peppermint essential oil on exhaustion time. We found that rats in a dose of 4, 40 μL/h had longer exhaustion time than those in a dose of 400 μL/h. In order to determine a more precise dose, four doses of peppermint oil (4, 16, 64, 256 μL/h) were selected. The exhaustion times of the control group, 4, 16, 64, and 256 μL/h groups were 318.3 ± 37.28 s, 312.8 ± 32.99 s, 419.1 ± 60.19 s, 392.7 ± 59.61 s, and 284.4 ± 13.20 s, respectively. The swimming exhaustion time of rats in the 16 μL/h dose group was significantly longer than that in the control group. The dosage of 16 μL/h was chosen for the investigation of the chronic effects 2-week inhalation of peppermint essential oil on fatigue and endurance performance.

### 2.5. Weight-Loaded Swimming Training Protocol

Rats were forced to swim with continuous weight bearing (approximately 3% of the body weight) for 2 weeks to establish an exercise-induced chronic fatigue model adapted from previous models [11]. In particular, the rats were subjected to a tail-loaded method using a lead ball (weighing approximately 3% of the rat’s body weight) and then placed in a circular plastic bucket (65 cm height, 50 cm diameter) for free swimming. The water depth was 40 cm with a water temperature of 34 ± 2 °C. Rats were removed from the water immediately after 90 min swimming or upon exhaustion, which was determined by a lack of swimming coordination and submersion of the rat’s nostrils for more than 10 s before resurfacing.

### 2.6. Exhaustive Forced Swimming Test

The forced swimming test (FST) was used to measure the exhaustion time of rats [14]. The animals were loaded with a lead block (about 5% of the body weight of rats) attached to their tails. The rats were placed in a round plastic bucket (65 cm in height, 50 cm in diameter) to swim freely after loading. The average water area was 320 cm^2^ per rat, the depth of water was 50 cm, and the water temperature was 34 ± 2 °C. The rats were removed from the water immediately after swimming to exhaustion, and the exhaustion time was recorded. The exhaustion was judged according to the criteria in the literature [15,16].

### 2.7. Body Mass Measurement and Grip Strength Test

The body mass of rats in each group was measured before exercise, one week after exercise, and two weeks after exercise. The maximum grip strength of rats was measured using a small animal grip strength meter (ZS-ZL, Beijing Zhongshi Dichuang Technology Development Co., Ltd., Beijing, China). The rats were gently lifted by their tails to create a 15° angle between their bodies and the mesh surface of the grip strength meter. Their forepaws were then allowed to grip the mesh surface and pull back while the grip strength was recorded. Each rat was measured three times, and the average value was calculated.

### 2.8. Elevated plus Maze (EPM) Test

EPM test was carried out using the elevated cross maze video analysis system (RD-1108-EPM-R1, Shanghai Yishu Information Technology Co., Ltd., Shanghai, China). The EPM experiment was carried out using the method described in the literature [17]. The video analysis system calculated the activity indicators: the number of times to enter the open arm (OE); the time to enter the open arm (OT); the number of times to enter the closed arm (CE); the time to enter the closed arm (CT); the percentage of time to stay in the open arm, OT% = OT/(OT + CT) × 100%; and the percentage of times entering the open arm, OE% = OE/(OE + CE) × 100%.

### 2.9. Novel Object Recognition (NOR)

NOR experiment was performed using the behavior video analysis system (JLBehv-OFG-4, Shanghai, China). Rats were placed in the behavioral laboratory for 2 h of adaptation before the experiment. The open field space was measured at (80 × 80 × 50 cm) with black wooden boards on the bottom and sides. NOR experiment was conducted according to the method described in the reference [18], which consisted of three stages: adaptation, training, and testing. During the adaptation phase, each rat was allowed to explore the open field without any objects for 10 min. In the training phase, all rats were allowed to explore the open field with two identical cubic wooden blocks (15 × 15 × 15 cm) located diagonally for 5 min while contact time and frequency were recorded. The training phase lasted 24 h, afterwhich the testing phase began. A cylindrical wooden block (15 cm in diameter, 15 cm in height) was substituted for one of the cubic blocks in the open field, and each rat was allowed to explore the open field with the novel object for 5 min. Contact time and frequency were recorded, and the novel object discrimination index (DI) was calculated as follows: DI = novel object exploration time/(novel object exploration time + original object exploration time) × 100% [19]. The open field was cleaned with 75% alcohol and tissue paper before each test.

### 2.10. Determination of Biochemical Parameters Related to Fatigue in Serum, Muscle, and Liver Tissues

After an exhaustive swimming test, the rats were anesthetized with a constant inflow of 2.5% isoflurane mixed with oxygen. Blood samples were collected from the rat heart apex and stored at room temperature for 20 min before centrifuged at 4 °C for 15 min (3500 r/min). The supernatant was collected for testing. The liver and gastrocnemius muscle tissues were collected and rapidly frozen in liquid nitrogen and then stored in a −80 °C freezer for testing.

The contents of glucose (GLU), blood urea nitrogen (BUN), lactic acid (LA), and lactate dehydrogenase (LDH) in serum; superoxide dismutase (SOD), malondialdehyde (MDA), reduced glutathione (GSH), and reduced glutathione peroxidase (GSH-PX) in skeletal muscles; and GSH, GSH-PX, xanthine oxidase (XOD), and catalase (CAT) in liver tissues were measured using commercial test kits according to the manufacturer’s instruction.

### 2.11. Statistical Analysis

The data were analyzed using GraphPad Prism 8.0, and the experimental data for each group were expressed as the mean ± standard error of the mean (SEM). Student’s *t*-test was used to compare the data between two groups, while one-way or two-way ANOVA was used to compare data among multiple groups. When variances were equal among groups, the Bonferroni method was used for comparison; when variances were unequal, the Tamhane T2 test was used. *p <* 0.05 was considered statistically significant for intergroup differences.

## 3. Results

### 3.1. Composition and Content of Peppermint Essential Oil

**Figure 2:**
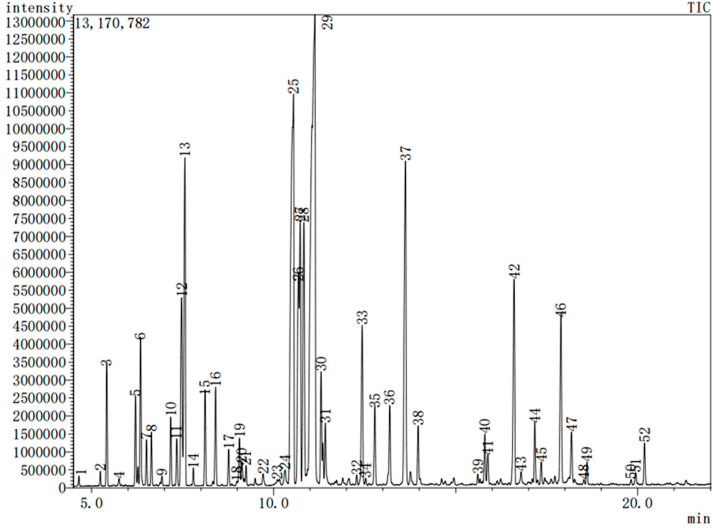
GC-MS chromatogram of peppermint essential oil.

Menthol, menthol ketone, menthol acetate, and menthol furan are the main components that constitute the characteristic aroma of peppermint essential oil, giving the essential oil a cool and high-quality mint aroma [6]. We used GC-MS to analyze and identify the volatile chemical components of the essential oil. The obtained chromatogram of peppermint essential oil showed 52 chemical components, accounting for 99.34% of the essential oil, as illustrated in Figure 2 and Table 1. Among them, there are 12 main components with a content of more than 1% in peppermint essential oil, which are menthol (37.01%), menthone (21.83%), 1,8-cineole (6.36%), L-menthol acetate (5.34%), (+)-menthone (4.71%), D-menthol (3.41%), β-Caryophyllene (2.66%), menthofuran (2.43%), menthone (1.94%), (R)-(+)-limonene (1.89%), β-Cyclopentene (1.62%), and β-Pinene (1.41%), accounting for 90.61% of the total composition of the essential oil.

### 3.2. Peppermint Essential Oil Treatment Had No Effect on Body Weight and Grip Strength in Rats

**Figure 3:**
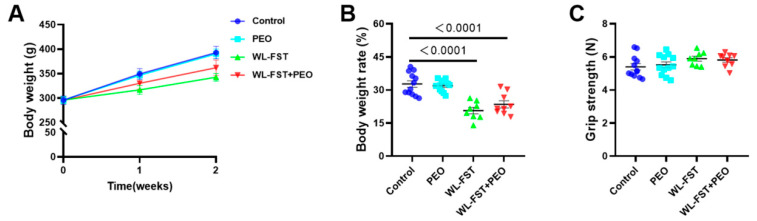
Effects of peppermint essential oil on body weight and grip strength of rats. (**A**,**B**) Body weight and weight change rate of rats during two weeks of exercise training (n = 8–12). (**C**) Measurement of grip strength after two weeks of exercise training (n = 8–12). Data are presented as mean ± SEM. Data were analyzed using one-way or two-way analysis of variance followed by the Bonferroni multiple comparison test. Values of 95%CI and effect size (Cohen’s d) are in Appendix A, Table A1.

After 2-week forced swimming, the body weight of rats dropped to 342.6 ± 7.8 g (WL-FST) and 362.0 ± 14.7 g (WL-FST+PEO) compared with the control (390.3 ± 12.2 g), with a significant difference in the rate of body weight change (Figure 3A,B). Grip strength measurement showed that there was no significant difference among the groups, 5.40 ± 0.19 N in the control, 5.52 ± 0.17 N in PEO, 5.80 ± 0.18 N in WL-FST, and 5.77 ± 0.13 N in WL-FST+PEO (Figure 3C). The results indicated that inhaling peppermint essential oil had no effect on muscular strength in endurance-trained rats.

### 3.3. Peppermint Essential Oil Extended the Time to Exhaustion

**Figure 4:**
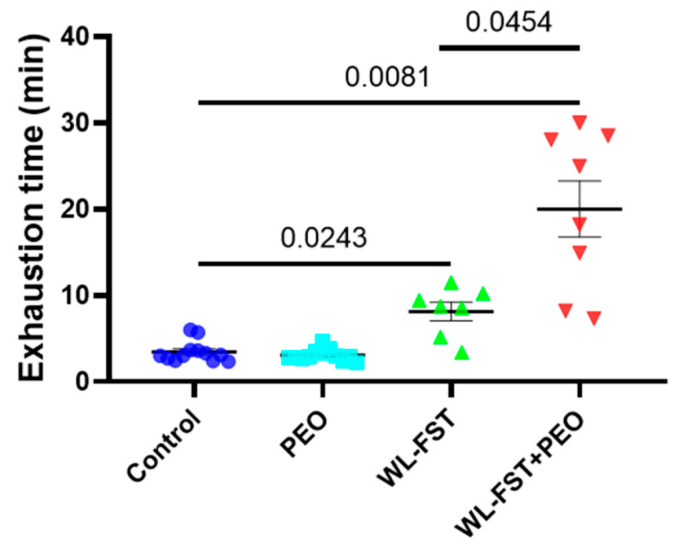
Mean swimming time to exhaustion in exhaustive swimming test following 2-week weight-loaded endurance exercise. n = 7–12. Data are presented as mean ± SEM. Data were analyzed using a one-way analysis of variance followed by the Bonferroni multiple comparison test. Values of 95%CI and effect size (Cohen’s d) are in Appendix A, Table A2.

As a measure for defining exercise performance, the mean swimming time until exhaustion was recorded in an exhaustive swimming test following 2-week endurance training. The mean swimming time until exhaustion in the WL-FST+PEO group was 18.55 ± 4.33 min, significantly prolonged compared with 8.06 ± 1.28 min in the WL-FST group (Figure 4). In addition, rats in WL-FST performed better than the control rats, indicating the effects of exercise training. However, unexercised rats who received essential oil inhalation for 2 weeks did not perform as well as the rats in WL-FST (Figure 4). The results suggest that inhalation of essential oil and exercise training synergistically improves exercise performance.

### 3.4. Effects of Peppermint Essential Oil on Energy Metabolites in Serum

**Figure 5:**
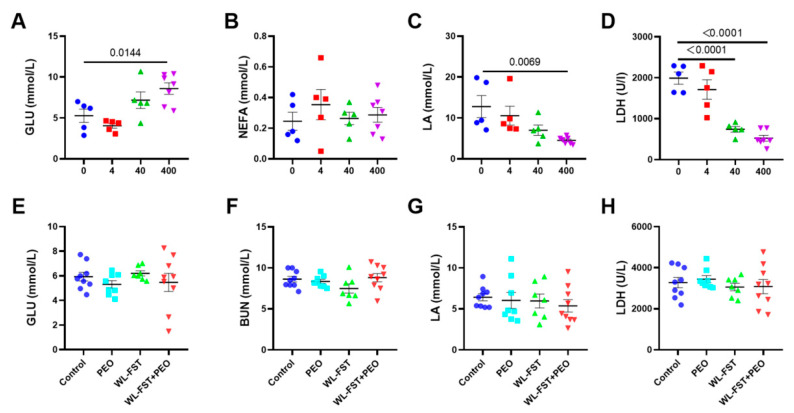
Changes in serum concentration of markers associated with energy metabolism and fatigue. (**A**–**D**): serum levels of GLU, NEFA, LA, and LDH in rats with acute exercise-induced fatigue following interventions with peppermint essential oil at different dosages (this investigation was the acute experiment intended to screen the optimal dose of peppermint oil for chronic experiment). (n = 5–7). (**E**–**H**): serum levels of GLU, BUN, LA, and LDH in rats with chronic exercise-induced fatigue (n = 7–10). Data are presented as mean ± SEM. Data were analyzed using one-way analysis of variance followed by the Bonferroni multiple comparison test. GLU, glucose; NEFA, nonestesterified fatty acid; LA, lactate acid; LDH, lactate dehydrogenase; BUN, blood urea nitrogen. Values of 95%CI and effect size (Cohen’s d) are in Appendix A, Table A3.

In the acute forced swimming experiment, the serum glucose levels of the control group and the low, medium, and high dose groups were 5.28 ± 0.81, 4.06 ± 0.31, 7.18 ± 1.01, and 8.60 ± 0.70 mmol/L, respectively. Compared with the control, the serum glucose levels in rats increased with increasing doses of inhaled peppermint essential oil, with a significant increase observed in the high-dose group (*p* = 0.0144) (Figure 5A).

The serum LA levels of the control group and the low, medium, and high dose groups were 12.80 ± 2.86, 10.57 ± 2.31, 7.02 ± 1.26, and 4.51 ± 0.31 mmol/L, respectively, with a significant reduction observed in the high dose group compared to the control group (*p* = 0.0069) (Figure 5C).

The serum LDH activity of the control group and the low, medium, and high dose groups were 1990.52 ± 147.08, 1712.412 ± 239.36, 741.86 ± 69.58, and 522.86 ± 70.47 U/L, respectively. As the dose of inhaled peppermint essential oil increased, the serum LDH activity in rats decreased, with a significant decrease in high-dose groups compared to the control group (*p* < 0.0001) (Figure 5D). These data suggest that peppermint essential oil can help to relieve fatigue.

In the chronic exercise-induced fatigue experiment, we chose peppermint essential oil at a dosage of 16 μL/h and did not observe a significant difference (*p* > 0.05) in the serum levels of GLU, BUN, LA, and LDH (Figure 5E–H) among different rat groups.

### 3.5. Effects of Peppermint Essential Oil on Redox Biomarkers in Skeletal Muscle and Liver Tissues

**Figure 6:**
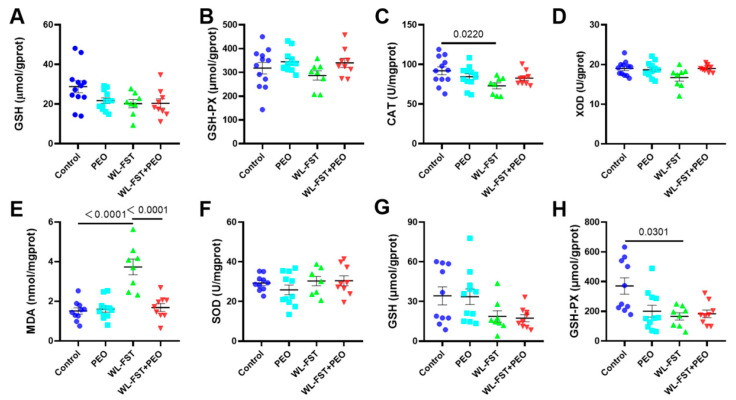
Effects of peppermint essential oil on redox biomarkers in liver and skeletal muscle tissues in rats. Redox biomarkers in liver and skeletal muscle tissues in rats were measured after 2-week period. (**A**–**D**) concentrations of GSH, GSH-PX, CAT, and XOD in rat liver tissues (n = 8–12). (**E**–**H**) concentrations of MDA, SOD, GSH, and GSH-PX in rat skeletal muscle tissues (n = 8–11). Data are presented as mean ± SEM. Data were analyzed using a one-way analysis of variance followed by the Bonferroni multiple comparison test. GSH, glutathione; GSH-PX, glutathione peroxidase; CAT, catalase; MDA, malondialdehyde; SOD, superoxide dismutase; XOD, xanthine oxidase. Values of 95%CI and effect size (Cohen’s d) are in Appendix A, Table A4.

The mean MDA content in skeletal muscle of rats in the control group, PEO group, WL-FST group, and WL-FST+PEO group were 1.51 ± 0.16 nmol/mgprot, 1.61 ± 0.16 nmol/mgprot, 3.73 ± 0.40 nmol/mgprot, and 1.68 ± 0.20 nmol/mgprot, respectively. Compared with the control, the content of MDA in the skeletal muscle of rats in the WL-FST group was significantly increased (*p* < 0.01), while that in the WL-FST+PEO group was significantly lower than that in the WL-FST group (*p* < 0.01) (Figure 6E). It suggests that the level of peroxidation in the skeletal muscle of rats after endurance exercise increased and caused oxidative damage. The oxidative damage was effectively prevented by peppermint essential oil treatment.

GSH Px content in skeletal muscle tissue of rats in the WL-FST group was lower than that in the control group (166.26 ± 24.27 vs. 370.76 ± 54.93, *p* < 0.05), but there was no significant difference between WL-FST+PEO group and WL-FST group (*p* > 0.05) (Figure 6H). The contents of SOD and GSH in the skeletal muscle of animals in each group had no significant difference (*p* > 0.05) (Figure 6F,G). In addition, there was no significant difference in the contents of GSH, GSH Px, CAT, and XOD in the liver tissues (*p* > 0.05) (Figure 6A–D), suggesting no significant changes in redox status.

### 3.6. Peppermint Essential Oil Had No Significant Effect on Cognitive Functions of Rats

**Figure 7:**
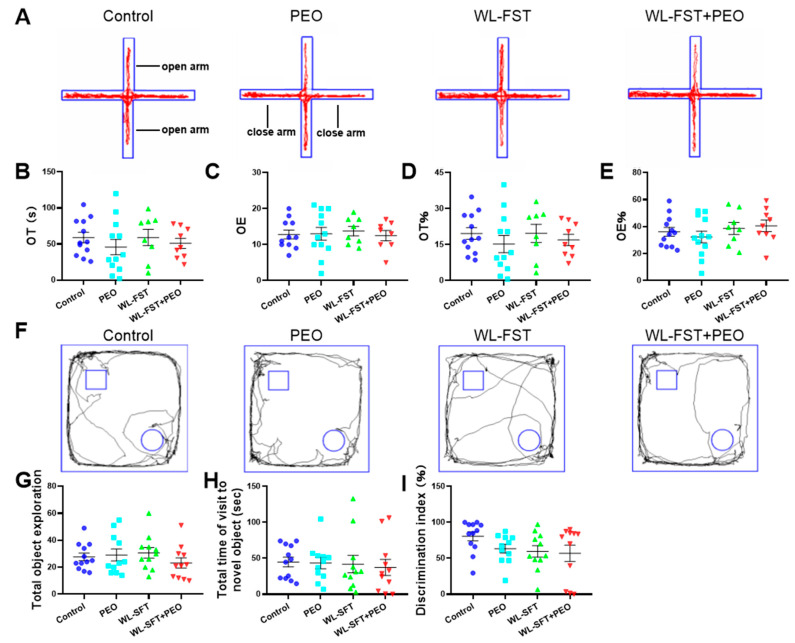
Effect of peppermint essential oil on cognitive functions in rats: (**A**) The movement of rats in elevated plus maze tests (n = 8–12); (**B**) the average time spent in the open arm; (**C**) the average number of entries into the open arm; (**D**) the average percentage of time spent in the open arm; (**E**) the average percentage of entries into the open arm; (**F**) the movement of rats in novel object recognition tests (n = 11–12); (**G**) the average total exploration times of objects; (**H**) the average of exploration times of the new objects; (**I**) the average novel object discrimination index values. One-way ANOVA was used for comparison between groups. The values are presented as mean ± SEM.

In the EPM experiment, the average time spent in the open arms of the control, PEO, WL-FST, and WL-FST+PEO groups of rats were 58.99 ± 7.35 s, 45.77 ± 10.72 s, 59.09 ± 11.36 s, and 50.88 ± 7.07 s, respectively. The average numbers of entries into the open arms were 12.82 ± 1.20, 13.00 ± 1.78, 13.75 ± 1.32, and 12.50 ± 1.40 times, respectively. The average percentage of time spent in the open arms was 19.66 ± 2.45% and 36.22 ± 3.31%, 15.26 ± 3.57%, 32.33 ± 4.30%, and 19.70 ± 3.79%, and the percentage of entries into the open arms were 36.22 ± 3.31%, 32.33 ± 4.30%, 38.68 ± 4.51%, and 40.61 ± 4.26%, respectively. These results indicated that 2-week weight-bearing swimming training did not produce significant mental fatigue as characterized by impaired cognitive functions, and peppermint essential oil had no significant impact on the cognitive ability of rats (Figure 7A–E).

In the NOR experiment, the average total exploration times of the control, PEO, WL-FST, and WL-FST+PEO groups of rats were 30.17 ± 2.98, 30.17 ± 4.50, 30.33 ± 4.11, and 29.50 ± 5.17 times, respectively. The average exploration times of the new object were 44.83 ± 6.72 s, 40.12 ± 7.94 s, 39.31 ± 11.44 s, and 34.27 ± 10.63 s, respectively. The average novel object DI values were 80.52 ± 6.53%, 64.46 ± 5.62%, 61.42 ± 7.46%, and 52.14 ± 11.48%, respectively (Figure 7F–I).

## 4. Discussion

In the present study, we investigated the anti-fatigue effects of peppermint essential oil on endurance exercise performance in rats subjected to a 2-week weigh-loaded forced swimming regimen. We found that 14-day inhalation of peppermint essential oil significantly prevented oxidative damage and extended the time to exhaustion in the exhaustive swimming test.

It is generally believed that fatigue acts as a protective mechanism from the deleterious effects of exercise; however, chronic fatigue due to prolonged exhaustion from physical activity may cause irreversible damage to the body’s metabolism and even compromise overall health. The relationship between physical exercise and fatigue has been the scope of interest of many researchers for more than a century and is very complex [4]. Exercise-induced metabolic alterations; the release of cytokines; an increase in oxidative stress; and the accumulation of metabolites, such as lactic acid or lactate dehydrogenase, leads to muscle fatigue. A number of natural bioactive herbs have been shown to have the ability to prevent and alleviate exercise-induced fatigue through various complex biological reactions. Among these, peppermint is one of the most famous natural herbs, with wide application in complementary medicine. Peppermint essential oil, which contains the major components menthol and menthone, has been applied as a dietary supplement, nutraceutical, or massage oil due to its antioxidant, anti-inflammatory, antibacterial, antispasmodic, and anti-fatigue effects [10,20,21,22]. It has also been shown to relieve exercise fatigue by inhibiting accumulated metabolites and enhancing antioxidant enzyme activity [23,24]. In the present study, we found that repeated inhalation of peppermint essential oil significantly reduced the elevated levels of malondialdehyde in skeletal muscles induced by 2-week exhaustive swimming in rats. Malondialdehyde (MDA) is a marker of lipid peroxidation, which can occur when free radicals damage cell membranes and lead to reactive oxygen species (ROS) production. Inflammation is also thought to be involved in the formation of reactive oxygen species (ROS) and lipid peroxidation. Peppermint essential oil has been shown to possess free radical-scavenging activity and anti-inflammatory properties. Thus, it could scavenge ROS and reduce the inflammation-mediated generation of ROS, and subsequently decrease MDA levels. However, there were no significant effects observed on redox biomarkers, such as SOD and GSH-px, and energy metabolites were not significantly affected. This may be because essential oil is administered through nasal inhalation, which differs from the effects observed when peppermint is administered orally or applied via massage. Interestingly, in acute forced swimming experiment which was performed to determine an optimal dose of peppermint essential oil, we observed that essential oil at medium (40 μL/h) and high dosages (400 μL/h) showed remarkable effects on energy metabolites, such as lactic acid and lactate dehydrogenase, but with limited effects on exercise performance. We finally chose an optimal dosage of 16 μL/h in chronic endurance exercise, which showed remarkable effects on improving exercise performance, while the effects on energy metabolites were limited.

In addition, a remarkable contrast in appearance and behavior was observed between the rats treated or untreated with essential oil. With an increase in the number of swimming days, rats without essential oil administration showed a series of fatigue symptoms such as lethargy, disheveled and withered fur, reduced appetite, weak physical condition, dull and lifeless eyes, and delayed response to external stimulation. The rats that inhaled peppermint essential oil before each load-bearing swimming training showed an improvement in their condition, exhibiting increased appetite and better physical condition with an increase in the number of exercise days. After swimming and exiting from the water, these rats were lively and active and were able to shake their bodies vigorously and quickly dry their fur through shaking.

Importantly, in the exhaustive swimming test, the endurance-trained rats receiving peppermint essential oil inhalation demonstrated a significantly extended length of swimming time to exhaustion compared to the exercised rats without essential oil treatment. Interestingly, the exercised rats performed better than the sedentary control, indicating the positive exercise training effects. Moreover, even the sedentary rats who received essential oil inhalation for 2 weeks did not perform as well as the exercised rats. These results suggest that peppermint essential oil may not primarily serve as an ergogenic aid to enhance physical performance. Instead, it appears to reinforce the effects of exercise training, potentially through mechanisms related to anti-fatigue or increasing pain tolerance.

Our data showed that peppermint essential oil administration remarkably enhanced exercise performance in rats subjected to 2-week exhaustive swimming, despite having limited effect on energy metabolic and redox biomarkers. This is significant because central fatigue, which originates in regions of the brain, is a major contributor to exercise-induced fatigue. This is consistent with the idea that exercise-induced fatigue involves primarily central or supraspinal mechanisms more than peripheral or muscle fatigue. Since endurance exercise induces proinflammatory cytokines that may cause changes in behavior by provoking feelings of fatigue, it is reasonable to assume that this increase in circulating proinflammatory signals during prolonged and strenuous exercise may contribute to exercise-induced fatigue [17]. Central fatigue, which is characterized by the inability of the central nervous system to efficiently drive motor neurons during intermittent or prolonged aerobic exercise, and peripheral or muscle fatigue resulting from biochemical changes in the exercising limb muscles, are both contributors to exercise-induced fatigue [17]. Previous research suggests that peppermint essential oil can protect the brain and nervous system, with its neuroactive compounds menthol and menthone inhibiting cholinesterase and binding to nicotine and GABAA receptors, resulting in increased neural activity. Continuous administration of peppermint essential oil significantly facilitates the elimination of mental fatigue [25]. Studies on humans have also shown potential cognitive benefits of peppermint, such as improving memory and reducing mental fatigue associated with extended cognitive task performance in healthy young men [25,26]. In this study, we did not find obvious effects of essential oil on cognitive performance, probably because 2-week weight-bearing swimming training was not enough to cause central fatigue that impairs cognitive function.

The effects of peppermint oil on exercise performance and fatigue in human studies have yielded inconsistent findings. Some researchers have reported increased performance and respiratory function parameters following inhalation or oral supplementation of peppermint essential oil in healthy young men [27,28]. Conversely, Shepherd et al. conducted a study using the same protocol of oral supplementation of peppermint oil and found that it had no effect on aerobic capacity and performance during a graded maximal exercise test [29]. Moreover, some studies reported that inhaling peppermint oil had no significant effects on exercise performance [30,31]. Of note, these studies testing the effect of peppermint essential oil were either conducted after a sedentary period when peppermint essential oil was supplemented or involved only a brief 10–15 min inhalation before the exercise session. In our small-sized preliminary study, five healthy young volunteers (Table S1) received peppermint inhalation during the recovery period after exhaustive cycling and reported feeling good, while peppermint essential oil did not result in significant changes in the physiological measurements and redox biomarkers (Figures S1 and S2, Table S2), which is consistent with previous human studies. These findings suggest that short-term use of peppermint essential oil may not be effective in improving exercise performance. As suggested by the present study, it seems that the continuous supplementation with peppermint essential oil can augment the positive effects of exercise through redox balancing and alleviating fatigue sensation or other mechanisms.

Prior research has demonstrated that inhalation of peppermint essential oils reduced the subjective feelings of fatigue and improved the feelings of refreshment [32], though physiological and biochemical measurements of exercise-induced fatigue or performance show insignificant changes. This is suggestive that the sensations of fatigue are largely independent of biological and physiological factors. Aromatherapy has been shown to positively influence exercisers’ experiences during both exercise and recovery periods, resulting in greater exercise satisfaction and adherence [33,34]. Given the combined biological and psychological beneficial effects of peppermint essential oil and both the physiological and mental nature of exercise fatigue, peppermint oil may represent an advantageous choice for enhancing exercise performance, particularly during endurance exercise. There has not been any current opinion from the World Anti-Doping Agency (WADA) regarding the inhalation of peppermint essential oil and its effects on exercise performance. The expert consensus regarding the use of menthol as an ergogenic aid for the Tokyo 2021 Olympic Games promotes the development of guidelines for its safe administration and encourages further research on the benefits of peppermint essential oil for endurance sports [35]. Further studies, with particular attention paid to participants’ perceived pleasantness of essential oils and their motivation towards exercise, will enable a more comprehensive understanding of the interaction between subjective feelings and physiological responses during exercise. This will contribute to the development of new strategies for improving exercise adherence and performance.

## 5. Conclusions

This study provides evidence that repeated inhalation of peppermint essential oil during exercise training can enhance the effects of endurance exercise training and improve physical performance, which is partly attributed to the antioxidant effects of peppermint essential oil.

## Figures and Tables

**Table 1:** Major components of peppermint essential oil identified by GC-MS.

Peak NO.	Components	CAS NO.	RT (min)	Area (%)
1	menthol	1490-04-6	11.13	37.01
2	menthone	14073-97-3	10.55	21.83
3	1,8-cineole	470-82-6	7.56	6.36
4	L-menthol acetate	16409-45-3	13.62	5.34
5	(+)-menthone	3391-87-5	10.73	4.71
6	D-menthol	15356-60-2	10.83	3.41
7	β-Caryophyllene	87-44-5	16.61	2.66
8	menthofuran	494-90-6	10.67	2.43
9	(+)-pulegone	89-82-7	12.43	1.94
10	(R)-(+)-limonene	5989-27-5	7.47	1.89
11	β-Cyclopentene	18252-44-3	17.89	1.62
12	β-Pinene	18172-67-3	6.34	1.41
				90.61

CAS No.: Chemical abstracts service registry number; RT: retention time; components with ≥1% of relative content (Area %) are listed.

## Data Availability

The data presented in this study are available in this article.

## Supplementary Materials

The following supporting information can be downloaded at: https://www.mdpi.com/article/10.3390/nu15112480/s1. Figure S1: Physiological variables before and after peppermint essential oil inhalation; Figure S2: Effect of peppermint essential oil on serum metabolites after exercise; Table S1: Socio-demographic characteristics of the study population; Table S2: Effect of peppermint essential oil on serum metabolites after exercise.

## Appendix A

**Table A1:** Effects of peppermint essential oil on body weight change rate.

Groups	Mean (SEM)	95% CI	Effect Size (Cohen’s d)		
			vs. PEO	vs. WL-FST	vs. WL-FST+PEO
Control	32.77 (1.47)	(29.52, 36.01)	0.20	2.61	1.86
PEO	31.97 (0.75)	(30.33, 33.62)	/	3.28	2.18
WL-FST	20.67 (1.46)	(17.22, 24.12)	/	/	−0.63
WL-FST+PEO	23.50 (1.46)	(19.77, 27.23)	/	/	/

**Table A2:** Time to exhaustion in exhaustive swimming test following 2-week weight-loaded endurance exercise.

Groups	Mean (SEM)	95% CI	Effect Size (Cohen’s d)		
			vs. PEO	vs. WL-FST	vs. WL-FST+PEO
Control	3.46 (0.35)	(2.69, 4.22)	0.40	−2.14	−2.53
PEO	3.07 (0.19)	(2.64, 3.49)	/	−2.44	−2.60
WL-FST	8.16 (1.09)	(5.51, 10.81)	/	/	−1.74
WL-FST+PEO	20.03 (3.25)	(12.34, 27.71)	/	/	/

**Table A3:** Changes in serum concentration of markers associated with energy metabolism and fatigue.

	Groups	Mean (SEM)	95% CI	Effect Size (Cohen’s d)		
				vs. 4	vs. 40	vs. 400
GLU	0	5.28 (0.81)	(3.03, 7.52)	0.89	−0.93	−1.81
	4	4.06 (0.31)	(3.21, 4.91)	/	−1.87	−3.25
	40	7.18 (1.01)	(4.38, 9.98)	/	/	−0.68
	400	8.60 (0.70)	(6.88, 10.31)	/	/	/
LA	0	12.80 (2.68)	(5.35, 20.24)	0.40	1.23	1.94
	4	10.57 (2.31)	(4.16, 16.98)	/	0.85	1.63
	40	7.02 (1.26)	(3.53, 10.51)	/	/	1.21
	400	4.51 (0.31)	(3.75, 5.28)	/	/	/
LDH	0	1990.52 (147.08)	(1582.16, 2376.98)	0.63	4.85	5.49
	4	1712.41 (239.36)	(1047.84, 2376.98)	/	2.46	2.97
	40	741.86 (69.58)	(548.67, 935.05)	/	/	1.27
	400	522.86 (70.47)	(350.42, 695.30)	/	/	//

**Table A4:** Effects of peppermint essential oil on redox biomarkers in liver and skeletal muscle tissues in rats.

	Groups	Mean (SEM)	95% CI	Effect Size (Cohen’s d)		
				vs. PEO	vs. WL-FST	vs. WL-FST+PEO
CAT	Control	92.12 (5.00)	(81.12, 103.11)	0.49	1.32	0.67
	PEO	84.61 (3.80)	(76.24, 92.97)	/	0.95	0.16
	WL-FST	73.08 (3.85)	(63.99, 82.18)	/	/	−0.68
	WL-FST+PEO	82.82 (3.02)	(75.85, 89.78)	/	/	/
MDA	Control	1.51 (0.16)	(0.15, 1.87)	−0.19	−2.55	−0.32
	PEO	1.61 (0.16)	(1.25, 1.96)	/	−2.43	−0.14
	WL-FST	3.73 (0.40)	(2.79, 4.68)	/	/	2.28
	WL-FST+PEO	1.68 (0.59)	(1.23, 2.14)	/	/	/
GSH-PX	Control	370.76 (54.93)	(246.49, 495.03)	1.10	1.55	1.39
	PEO	201.12 (39.68)	(112.71, 289.54)	/	0.33	0.16
	WL-FST	166.26 (24.73)	(108.86, 223.66)	/	/	−0.12
	WL-FST+PEO	183.76 (25.57)	(124.81, 242.72)	/	/	/

## References

[1] Azizbeigi K., Stannard S.R., Atashak S., Haghighi M.M. (2014). Antioxidant Enzymes and Oxidative Stress Adaptation to Exercise Training: Comparison of Endurance, Resistance, and Concurrent Training in Untrained Males. J. Exerc. Sci. Fit..

[2] Lee J.-S., Kim H.-G., Han J.-M., Kim Y.-A., Son C.-G. (2015). Anti-Fatigue Effect of Myelophil in a Chronic Forced Exercise Mouse Model. Eur. J. Pharmacol..

[3] Noakes T.D. (2012). Fatigue Is a Brain-Derived Emotion That Regulates the Exercise Behavior to Ensure the Protection of Whole Body Homeostasis. Front. Physiol..

[4] Ament W., Verkerke G.J. (2009). Exercise and Fatigue. Sports Med..

[5] Bowtell J., Kelly V. (2019). Fruit-Derived Polyphenol Supplementation for Athlete Recovery and Performance. Sports Med..

[6] Liu Y., Shen X., Sha M., Feng Z., Liu Y. (2023). Natural Bioactive Flavonoids as Promising Agents in Alleviating Exercise-Induced Fatigue. Food Biosci..

[7] Luo C., Wei X., Song J., Xu X., Huang H., Fan S., Zhang D., Han L., Lin J. (2022). Interactions between Gut Microbiota and Polyphenols: New Insights into the Treatment of Fatigue. Molecules.

[8] Bastaki S.M., Adeghate E., Amir N., Ojha S., Oz M. (2018). Menthol Inhibits Oxidative Stress and Inflammation in Acetic Acid-Induced Colitis in Rat Colonic Mucosa. Am. J. Transl. Res..

[9] Mani Badal R., Badal D., Badal P., Khare A., Shrivastava J., Kumar V. (2011). Pharmacological Action of Mentha Piperita on Lipid Profile in Fructose-Fed Rats. Iran J. Pharm. Res..

[10] Zhao H., Ren S., Yang H., Tang S., Guo C., Liu M., Tao Q., Ming T., Xu H. (2022). Peppermint Essential Oil: Its Phytochemistry, Biological Activity, Pharmacological Effect and Application. Biomed. Pharmacother..

[11] Lin T.-C., Wang S.-H., Huan C.-C., Lai Y.-C., Song T.-Y., Tsai M.-S. (2018). Anti-Fatigue, Antioxidation, and Anti- Inflammatory Effects of Eucalyptus Oil Aromatherapy in Swimming-Exercised Rats. Chin. J. Physiol..

[12] Umezu T., Sakata A., Ito H. (2001). Ambulation-Promoting Effect of Peppermint Oil and Identification of Its Active Constituents. Pharmacol. Biochem. Behav..

[13] Chumpitazi B.P., Kearns G., Shulman R.J. (2018). Review Article: The Physiologic Effects and Safety of Peppermint Oil and Its Efficacy in Irritable Bowel Syndrome and Other Functional Disorders. Aliment. Pharmacol. Ther..

[14] Orhan C., Gencoglu H., Tuzcu M., Sahin N., Ojalvo S.P., Sylla S., Komorowski J.R., Sahin K. (2022). Maca Could Improve Endurance Capacity Possibly by Increasing Mitochondrial Biogenesis Pathways and Antioxidant Response in Exercised Rats. J. Food Biochem..

[15] Yan K., Gao H., Liu X., Zhao Z., Gao B., Zhang L. (2022). Establishment and Identification of an Animal Model of Long-Term Exercise-Induced Fatigue. Front. Endocrinol..

[16] Tanaka M., Nakamura F., Mizokawa S., Matsumura A., Nozaki S., Watanabe Y. (2003). Establishment and Assessment of a Rat Model of Fatigue. Neurosci. Lett..

[17] Proschinger S., Freese J. (2019). Neuroimmunological and Neuroenergetic Aspects in Exercise-Induced Fatigue. Exerc. Immunol. Rev..

[18] Gáll Z., Kelemen K., Tolokán A., Zolcseak I., Sável I., Bod R., Ferencz E., Vancea S., Urkon M., Kolcsár M. (2022). Anticonvulsant Action and Long-Term Effects of Chronic Cannabidiol Treatment in the Rat Pentylenetetrazole-Kindling Model of Epilepsy. Biomedicines.

[19] Pezze M.A., Marshall H.J., Fone K.C.F., Cassaday H.J. (2015). Dopamine D1 Receptor Stimulation Modulates the Formation and Retrieval of Novel Object Recognition Memory: Role of the Prelimbic Cortex. Eur. Neuropsychopharmacol..

[20] Saqib S., Ullah F., Naeem M., Younas M., Ayaz A., Ali S., Zaman W. (2022). Mentha: Nutritional and Health Attributes to Treat Various Ailments Including Cardiovascular Diseases. Molecules.

[21] Golestani M.R., Rad M., Bassami M., Afkhami-Goli A. (2015). Analysis and Evaluation of Antibacterial Effects of New Herbal Formulas, AP-001 and AP-002, against Escherichia Coli O157:H7. Life Sci..

[22] Zhang Z., Engel M.A., Koch E., Reeh P.W., Khalil M. (2021). Menthacarin Induces Calcium Ion Influx in Sensory Neurons, Macrophages and Colonic Organoids of Mice. Life Sci..

[23] Li Z., Wu F., Shao H., Zhang Y., Fan A., Li F. (2017). Does the Fragrance of Essential Oils Alleviate the Fatigue Induced by Exercise? A Biochemical Indicator Test in Rats. Evid. Based Complement. Altern. Med..

[24] Sönmez G., Çolak M., Sönmez S., Schoenfeld B. (2010). Effects of Oral Supplementation of Mint Extract on Muscle Pain and Blood Lactate. Biomed. Hum. Kinet..

[25] Kennedy D., Okello E., Chazot P., Howes M.-J., Ohiomokhare S., Jackson P., Haskell-Ramsay C., Khan J., Forster J., Wightman E. (2018). Volatile Terpenes and Brain Function: Investigation of the Cognitive and Mood Effects of *Mentha × Piperita* L. Essential Oil with In Vitro Properties Relevant to Central Nervous System Function. Nutrients.

[26] Umezu T. (2013). Evaluation of Central Nervous System Acting Effects of Plant-Derived Essential Oils Using Ambulatory Activity in Mice. Pharmacol. Pharm..

[27] Meamarbashi A., Rajabi A. (2013). The Effects of Peppermint on Exercise Performance. J. Int. Soc. Sport. Nutr..

[28] Jaradat N.A., Al Zabadi H., Rahhal B., Hussein A.M.A., Mahmoud J.S., Mansour B., Khasati A.I., Issa A. (2016). The Effect of Inhalation of Citrus Sinensis Flowers and Mentha Spicata Leave Essential Oils on Lung Function and Exercise Performance: A Quasi-Experimental Uncontrolled before-and-after Study. J. Int. Soc. Sports Nutr..

[29] Shepherd K., Peart D.J. (2017). Aerobic Capacity Is Not Improved Following 10-Day Supplementation with Peppermint Essential Oil. Appl. Physiol. Nutr. Metab..

[30] Richmond S., Brammer C., Sosa A. (2016). The Effects of Peppermint Inhalation on Broad Jump and Vertical Leap. Med. Sci. Sports Exerc..

[31] Pournemati P., Azarbayjani M.A., Rezaee M.B., Ziaee V., Pournemati P. (2009). The Effect of Inhaling Peppermint Odor and Ethanol in Women Athletes. Bratisl. Med. J..

[32] Ohata M., Zhou L., Ando S., Kaneko S., Osada K., Yada Y. (2022). Application of Integrative Physiological Approach to Evaluate Human Physiological Responses to the Inhalation of Essential Oils of Japanese Citrus Fruits Iyokan (*Citrus Iyo*) and Yuzu (*Citrus Junos*). Biosci. Biotechnol. Biochem..

[33] Kwon S., Ahn J., Jeon H. (2020). Can Aromatherapy Make People Feel Better Throughout Exercise?. Int. J. Environ. Res. Public Health.

[34] Köteles F., Babulka P., Szemerszky R., Dömötör Z., Boros S. (2018). Inhaled Peppermint, Rosemary and Eucalyptus Essential Oils Do Not Change Spirometry in Healthy Individuals. Physiol. Behav..

[35] Barwood M.J., Gibson O.R., Gillis D.J., Jeffries O., Morris N.B., Pearce J., Ross M.L., Stevens C., Rinaldi K., Kounalakis S.N. (2020). Menthol as an Ergogenic Aid for the Tokyo 2021 Olympic Games: An Expert-Led Consensus Statement Using the Modified Delphi Method. Sports Med..

