# Supplementary material for: Repeated Inhalation of Peppermint Essential Oil Improves Exercise Performance in Endurance-Trained Rats
Source: Nutrients. 2023 May 26;15(11):2480. doi: 10.3390/nu15112480 (PMC10254935; doi:10.3390/nu15112480)
Supplement: Supplementary file 1 [file nutrients-15-02480-s001.zip › nutrients-2384170-supplementary.pdf]

Preliminary data of a small-sized preliminary human study

Participants

This was conducted according to the Declaration of Helsinki principles and was approved by the Human Research Ethics Committee of Fourth Military Medical University. Ten healthy and physically active young students (aged 22.0 ± 1.5 years, 7 male and 3 female) were recruited. Before the study, they were informed about the procedures and signed a written consent form. Their characteristics are in Table S1.

Table S1. Socio-demographic characteristics of the study population.

Group	Age	Height (cm)	Weight (kg)	BMI (kg/m^2)	Number (male/female)
Control	22.0±1.0	179.8±2.8	73.2±0.61	22.7±0.5	5/0
Peppermint essential oil	22.2±0.5	173.5±3.5	64.9±6.9	21.3±1.4	2/3

Study design

The approach is single-blinded. All participants were taking exercise test on cycle ergometer (939 novo, Monark, Sweden) twice with 1-hour recovery period in between. During the recovery, participants in the essential oil group received inhalation of peppermint essential oil for 5 min. Those in the control group just took a passive recovery.

Heart rate (HR) was monitored continuously using a HR belt (HRM-PRO, Garmin, Switzerland). The exercise protocol: warm-up for 2 min unloaded (0 W) exercise, then increased 15 W/min. The speed was set at 55-65 r/min until exhaustion followed by 3-min unloaded cycling. Exhaustion criteria: (1) heart rate exceeds 90% of maximum heart rate; (2) oxygen consumption reaches a plateau and starts to decline; (3) cycling speed drops below 55 r/min; (4) the participant shows painful facial expression or ask to stop the test. If 3 of the criteria are met, it will be considered exhaustion.

Blood was sampled from median cubital vein before, 0, 5, 15, and 60 min after cycling exercise using potassium ethylenediaminetetraacetic tubes. Samples were allowed to stand at room temperature for 20 min before centrifuged (15 min, 1600g, at 4 °C). Serum (supernatant) was extracted and stored at −80 °C without freeze–thaw cycles until assayed. Blood glucose was measured using a glucose meter (Life-Scan, Milpitas, CA, USA) once blood samples were collected.

Results

Physiological variables and biochemical measurements before and after peppermint essential oil inhalation are presented in Figures S1-2.

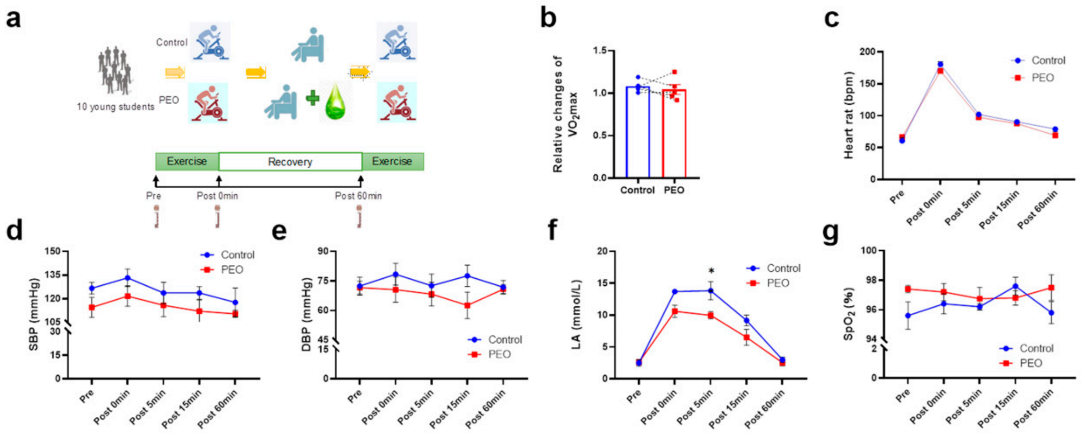

Figure S1. Physiological variables before and after peppermint essential oil inhalation. a. Protocol of pilot human study to examine the effects of peppermint essential on the recovery from exhaustive exercise. b. Relative changes of VO_2max between two sessions of exercise; c-g. Changes of heart rate, blood pressure, LA, and SpO_2 measured at different time points. n=5 per group, *$P<0.05$, the values are presented as mean ± SEM. In b, t-test was used for comparison between groups; in c-g, two-way ANOVA was used for comparison between groups. VO_2max, maximal oxygen uptake volume; SBP, systolic blood pressure; DBP, diastolic blood pressure; LA, lactic acid; SpO_2, oxygen saturation of peripheral tissues.

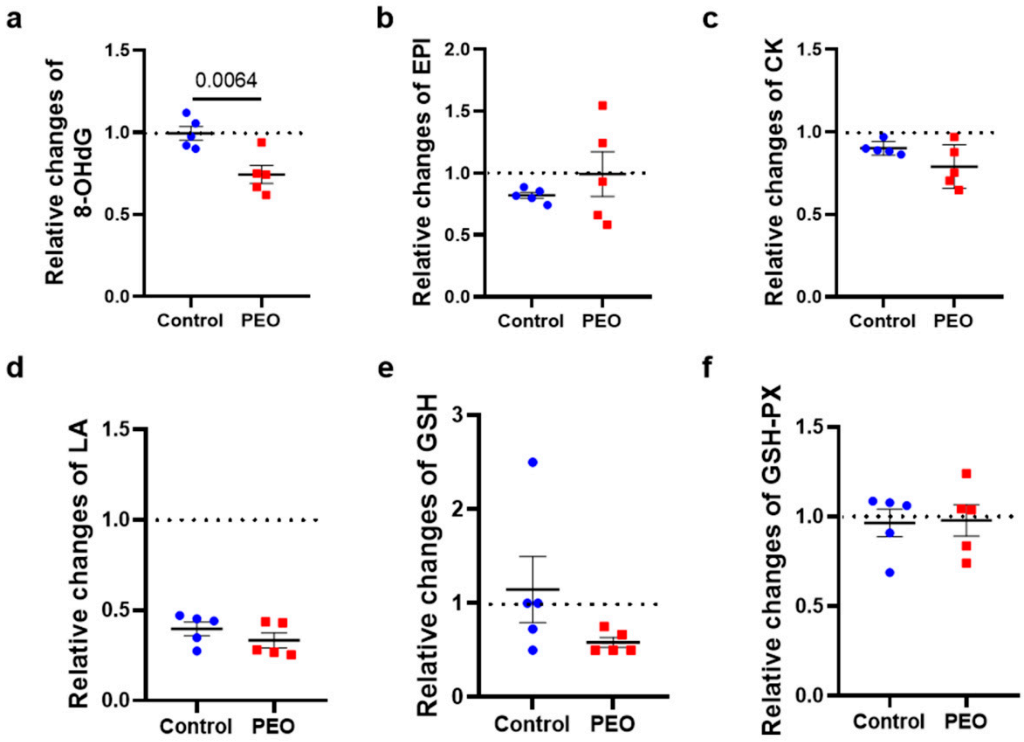

Figure S2. Effect of peppermint essential oil on serum metabolites after exercise. a-f: Relative changes of 8-OHdG, EPI, CK, LA, GSH and GSH-PX in serum between 60 minutes after exercise and immediately after exercise. n=5 per group, the values are presented as mean ± SEM. T-test was used for comparison between groups. 8-OHdG, 8-hydroxyguanosine; EPI, epinephrine; CK, creatine kinase; LA, lactate acid; GSH, glutathione; GSH-PX, glutathione peroxidase. Values of 95%CI and effect size (Cohen's d) are in Table S2.

Table S2. Effect of peppermint essential oil on serum metabolites after exercise

Relative changes	Control		PEO		Effect size (Cohen's d)
	Mean (SEM)	95 CI%	Mean (SEM)	95 CI%	
8-OHdG	1.00 (0.04)	(0.88, 1.11)	0.74 (0.05)	(0.59, 0.90)	2.32
EPI	0.82 (0.02)	(0.75, 0.89)	0.99 (0.18)	(0.49, 1.49)	-0.60
CK	0.90 (0.02)	(0.85, 0.95)	0.79 (0.06)	(0.63, 0.95)	1.14
LA	0.39 (0.03)	(0.29, 0.50)	0.33 (0.04)	(0.22, 0.45)	0.73
GSH	1.15 (0.35)	(0.17, 2.12)	0.58 (0.05)	(0.44, 0.73)	1.00
GSH-PX	0.96 (0.08)	(0.75, 1.18)	0.98 (0.09)	(0.74, 1.22)	-0.08

Informed Consent Statement: Informed consent was obtained from all subjects involved in this preliminary study.
